# Acute and Sub-Acute Toxicity Evaluation of the Methanolic Extract of *Alstonia scholaris* Stem Bark

**DOI:** 10.3390/medsci4010004

**Published:** 2016-03-08

**Authors:** Idris Bello, Abdulmenem Suliman Bakkouri, Yasser M. Tabana, Bassel Al-Hindi, Majed Ahmed Al-Mansoub, Roziahanim Mahmud, Mohd. Zaini Asmawi

**Affiliations:** 1 Department of Pharmacology, School of Pharmaceutical Sciences, University Sains Malaysia (USM), 11800 Pulau Pinang, Malaysia; idrisbello1@yahoo.com (I.B.); yasser.tabana@hotmail.com (Y.M.T.); basselalhindi@yahoo.com (B.A.-H.); madjed_25@yahoo.fr (M.A.A.-M.); 2 Department of Pharmaceutical Chemistry, School of Pharmaceutical Sciences, University Sains Malaysia (USM), 11800 Pulau Pinang, Malaysia; abdo_bakory@yahoo.com (A.S.B.); rozi@usm.my (R.M.)

**Keywords:** acute, *Alstonia scholaris*, sub-acute, toxicity, herb, plant, extract

## Abstract

*Alstonia scholaris* has been used by traditional medicine practitioners since the medieval ages for the treatment of diseases. The aim of this research was to evaluate the acute and sub-acute oral toxicity of its methanolic extract. The acute toxicity test was conducted using Sprague Dawley (SD) rats. The methanolic extract of *Alstonia scholaris* stem bark (ASME) was administrated in a single dose of 2000 mg/kg via oral gavage; and the animals were observed for any behavioral changes or mortality. In the sub-acute toxicity study, SD rats received three doses of ASME (250, 500 and 1000 mg/kg) for 28 days via oral gavage. During these 28 days of treatment, the rats were observed weekly for toxicity symptoms. Following the 28-day treatment, the rats were sacrificed for hematological, biochemical and histopathology studies. In the acute toxicity study, *Alstonia scholaris* was found to be non-toxic at a dose of 2000 mg/kg b.w. In the sub-acute toxicity study, significant variations in body weight, hematological and biochemical parameters were observed in the experimental groups at the dose of 500 and 1000 mg/kg with the death of two female rats being recorded at the highest dose (1000 mg/kg b.w.). Histopathological studies revealed slight degeneration (lesion) and centrilobular necrosis in the liver, which was most expressed in the highest-dose group. These results demonstrate that, while a single dose and short term oral intake of *Alstonia scholaris* bark extract caused no toxicity up to a dose of 2000 mg/kg b.w., toxic effects manifested in the long term treatment at the highest dose (500 and 1000 mg/kg). The long-term toxic effect was found to be associated with alterations in hematological compositions and end-organ damage to the liver. Thus, prolonged use of high doses of ASME orally should be discouraged and lower doses encouraged.

## 1. Introduction

Pharmacological evaluation of medicinal plants has recently witnessed a growing interest amongst researchers worldwide. Research on the therapeutic potential of plants has surged over the years, with volumes of scientifically documented information showing considerable potential for medicinal plants to be used in the treatment of several diseases [1]. However, while voluminous pharmacological studies have been conducted to ascertain the subjective traditional uses of various medicinal plants, very few plants have been thoroughly evaluated for their detrimental effect. Reports of efficacy are, by far, more numerous than those on toxicity [2,3]. There is, therefore, a need to further the investigation of herbal remedies and phytochemicals to incorporate the observations of short and long-term toxicity manifestations and to ensure effectual open communication of such findings.

*Alstonia scholaris*, a species of the family Apocyanaceae, has been widely studied for its numerous pharmacological properties [4]. It is native to the Indian subcontinent and Southeast Asia. *A. scholaris* has been investigated for its anti-inflammatory, analgesic [5], antidiabetic, antihyperlipidemic [6], anti-malarial [7], and anti-microbial effects [8], in addition to it theraputic potential against other diseases. Boiled decoctions of this herb have been reported to treat several diseases, such as asthma, hypertension, lung cancer and pneumonia; and also as remedies for fever [9,10,11]. The present study evaluates the acute and subacute oral toxicity of the methanol extract of *Alstonia scholaris* in experimental animals.

## 2. Experimental Section

### 2.1. Experimental Animals

Sprague Dawley (SD) rats, weighing 160–180 g, were obtained from the Animal Research and Service Center (ARSC) at Universiti Sains Malaysia (USM), Penang, Malaysia. The rats were kept in the Animal Transit Room at the School of Pharmaceutical Sciences, Universiti Sains Malaysia, where they were acclimatized to standard laboratory conditions for 7 days. Animals were fed standard rat diet *ad libitum* and were allowed free access to water. An approval was obtained from the Animal Ethics Committee, Universiti Sains Malaysia, Penang, Malaysia. The institutional animal ethical guidelines were strictly observed.

### 2.2. Plant Materials/Extract Preparation

The bark and leaves of *Alstonia scholaris* (*AS*) were sourced in 22 September 2013, from a forest in Penang, Malaysia, at the following coordinates (5.4246°N, 100.2689°E). The leaves were deposited at USM Herbarium for authentication (Specimen voucher registration No. 11479). Next, about 2.5 kg of air-dried, powdered stem barks of *Alstonia scholaris* (*AS*) were defatted with petroleum ether (60–80 °C ) to remove fat, latex and non-polar compounds of high molecular weights. The defatted plant residues were extracted by maceration in methanol for 24 h, with intermittent stirring at 45 °C, to obtain the methanol extract (ASME). The solvent was regularly changed until no coloration was observed. The collected extract was filtered through Whatman filter paper (No. 1). Lastly, the filtrate was concentrated in vacuum using rotary evaporator; and the concentrated extract was dried using a freeze dryer followed by incubation in an oven (45 °C).

### 2.3. Oral Acute Toxicity Study: Experimental Design

**Figure 1:**
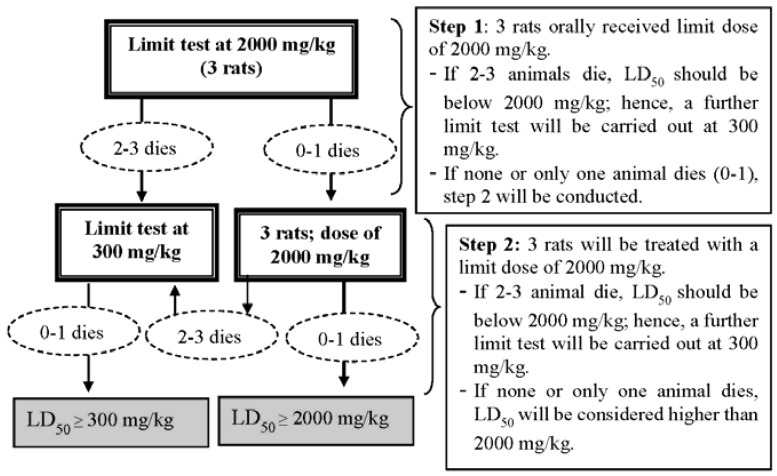
Acute toxicity chart flow.

The acute oral toxicity study was sanctioned to be conducted in compliance with OECD guideline 423, which stipulate the use of only three animals (OECD 423, Paragraph 23) [12,13]. Three of the test animals were fasted overnight (~12 h) and weighed. Test doses of *Alstonia* *scholaris* methanolic extract (ASME) were calculated in relation to the body weight of every fasted animal; and administered via oral gavage at 2000 mg/kg (Figure 1). The animals were regularly and individually observed for behavioral changes and general toxicity signs after dosing for the first 24 h, with special attention being given during the first 4 h. Thereafter, observation was continued daily for a total of 14 days [14].

### 2.4. Oral Sub-Acute Toxicity Study (28 Days)

#### 2.4.1. Procedure

The study was conducted in compliance with OECD guidelines No. 407. The experimental animals were divided into four groups of 10 rats each. Each group was made up of five, 9–11 week-old, nulliparous female rats (160–180 g b.w.); and 5 male (180–200 g b.w.) rats in separate cages. The groups were treated daily with three doses of ASME (250, 500 and 1000 mg/kg b.w.) for 28 days [15,16]. All treatments were administered via oral gavage.

#### 2.4.2. Observation

Clinical signs were observed at least twice a day during the 28-day treatment period. Body weights were measured once a week. On the 29th day, the animals were fasted overnight and blood samples collected via cardiac puncture. Vital body organs were dissected, cleansed of adhering tissues and rinsed in normal saline before their weights were measured. The kidneys and livers were immediately stored in 10% paraffin for histology. Paraffin sections were made and stained with hematoxylin and eosin for a thorough histopathological study [17,18]. Hematological analysis of the blood samples was performed using an automatic hematology analyzer. The parameters which were evaluated included: red blood cells (RBC) count; hemoglobin (Hb); mean corpuscular volume (MCV); mean corpuscular hemoglobin (MCH); mean corpuscular hemoglobin concentration (MCHC); platelets (PLT); leukocytes (WBC) count; and neutrophils, eosinophils, basophils, lymphocytes and monocytes counts. For biochemical analysis purposes, the blood samples were centrifuged at 3000 rpm for 15 min. Diagnostic kits were used to evaluate these parameters, which included the serum levels of total proteins (TP), bilirubin, alanine transaminase (ALT), aspartate transaminase (AST), alkaline phosphate (ALP), creatinine, and albumin (ALB); and the rats’ lipid profiles, *i.e.*, the levels of high density lipoproteins (HDL), low density lipoproteins (LDL), total cholesterol (TC) and total glycerol (TG). Histopathological examination was also conducted on the liver of the treated control groups [19,20]. Reference ranges for comparison are contingent on the method of analysis, animal species used and other experimental factors. Thus, in this study, the values obtained for the control group were considered as the reference values; and statistical analysis was conducted against the control group.

#### 2.4.3. Statistical Analysis

Results were expressed as the mean ± S.E.M. Statistical analysis was performed using version 21 of the IBM-SPSS statistical program (IBM Corp., Armonk, NY, USA). One-way ANOVA was used followed by Dunnett’s Test for parametric multiple comparisons between the control and the treatment groups. Differences were considered significant when the *p* value was less than 0.05 (*p* < 0.05).

## 3. Results

### 3.1. Acute Oral Toxicity Effects of Alstonia scholaris on Female SD Rats

There were no animal deaths in the first set of three female rats receiving 2000 mg/kg of *Alstonia scholaris* methanolic extract. No sign of toxicity was observed in the wellness parameters during the 14-day observation period. A similar observation was made in the second set of female rats treated with 2000 mg/kg of the extract. Therefore, the approximate acute lethal dose (LD_50_) of *Alstonia scholaris* extract in female rats was estimated to be higher than 2000 mg/kg.

### 3.2. Sub-Acute (28 Days) Oral Toxicity Effects of Alstonia scholaris on Male and Female SD Rats

#### 3.2.1. Effect of Oral Administration of *Alstonia scholaris* Extract on General Behavior

In the sub-acute toxicity study, both the male and female rats administered with 250 mg/kg b.w. of *A. scholaris* extract did not exhibit symptoms of toxicity. However, the male rats that received 1000 mg/kg of the extract showed signs of lethargy, and weakness; and abnormally slow motor and reflex activities. The symptoms of toxicity started manifesting around days 19–21 and days 25–26, respectively, in the groups treated with the 1000 and 500 mg/kg doses. Mortality and changes in respiratory rhythm and fur patterns were not observed during the 28-day experimental period in the aforementioned groups.

However, in the female rats, physical manifestation of toxicity symptoms occurred on days 20, 21 and days 14–16, respectively, in the groups receiving 500 and 1000 mg/kg b.w. of the extract. The toxic symptoms observed included lethargy, self-isolation, heavy breathing, changes in fur patterns, and abnormally slow motor and reflex activities. Overall, the toxic symptoms were most pronounced in the female group treated with the highest dose; and while no deaths were recorded in any of the male groups throughout 28 days of treatment, two of the female rats in the 1000 mg/kg group died on the 24th and the 25th days of the experiment.

#### 3.2.2. Effect of Oral Administration of *Alstonia scholaris* Extract on Body and Organs Weights

**Figure 2:**
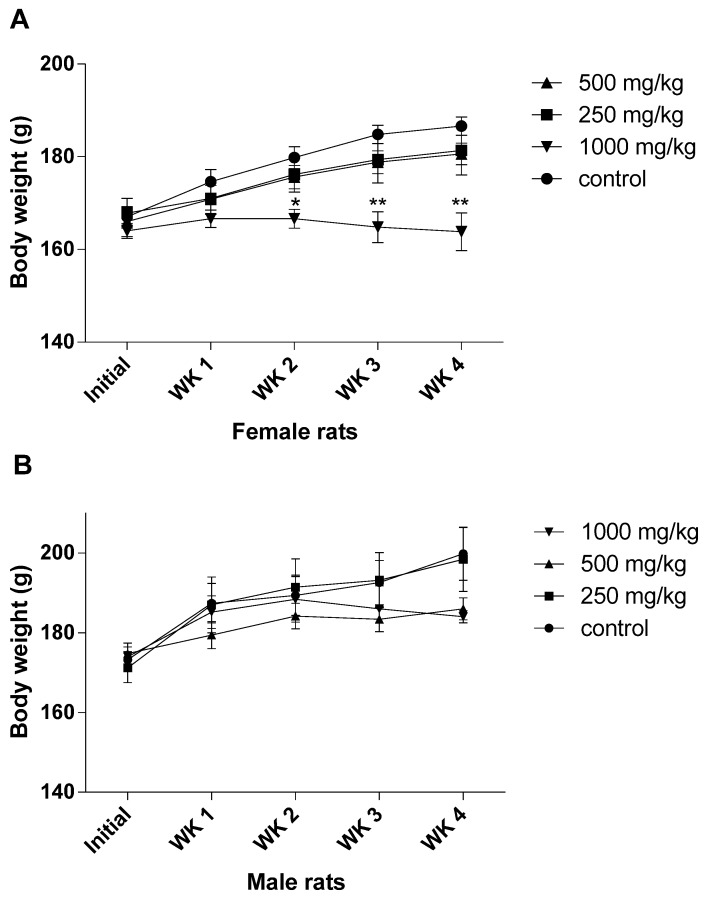
Initial and weekly (WK) body weight measurements (g) of the male (**A**) and female (**B**) rats in the sub-acute toxicity study of the methanolic extract of *Alstonia scholaris*. Results were expressed as the mean ± S.E.M. of 5 rats. (Significantly different from the control; * *p* < 0.05; ** *p* < 0.01).

The body weights and body weight gain of both the female and male rats treated with ASME doses (250, 500 and 1000 mg/kg b.w.) are presented in Figure 2. The female rats showed decreases in body weight compared with the control group, which were significant in the female rats receiving the extract at a dose of 1000 mg/kg b.w. of ASME starting from the 2nd week (*p* < 0.05) until the end of the experimentation period (*p* < 0.01). Moreover, after 28 days of treatment, the female rats which received 1000 mg/kg showed lower mean body weight compared with their initial weights. The male rats also showed decreased body weights in comparison with the control in a non-significant fashion.

The female rats’ organ weights are shown in Table 1. The rats treated with the extract at the doses of 500 and 1000 mg/kg b.w. had lung weights significantly (*p* < 0.05) lower than those of the control. Furthermore, significant decreases in the weights of the pancreas and the heart were observed in the groups treated with 500 and 100 mg/kg of ASME compared with the control group (*p* < 0.05). As Table 2 demonstrates, the male rats treated with 500 and 1000 mg/kg b.w. of the extract showed significant decreases in the weights of the lungs as well (*p* < 0.05). The mean organ weight of the spleen was also shown to be reduced in the 500 mg/kg group in a non-significant fashion. No significant differences were observed in the weights of the other organs compared to their respective control measurements.

#### 3.2.3. Effect of Oral Administration of *Alstonia scholaris* Extract on Serum Electrolytes Levels

Examination of serum electrolytes did not show much variation between the treated female (Table 3) or male (Table 4) groups and their respective control groups: sodium, potassium, chloride, urea, uric acid, calcium and phosphate concentrations in the treated groups were not significantly different from those in the control group.

#### 3.2.4. Effect of Oral Administration of *Alstonia scholaris* Extracts on Serum Biochemical Parameters

Alkaline phosphatase was significantly increased in all of the female treated groups (Table 5). Significant increases in the levels of aspartate transaminase (500 and 1000 mg/kg) and alanine transaminase (250 and 1000 mg/kg) were also observed (*p* < 0.01). Moreover, a mild increase in serum bilirubin levels was observed in the rats treated with 1000 mg/kg. In the male treated rats, ALT levels were significantly (*p* < 0.05) increased in all treated groups (Table 6). Increased AST and ALP levels were also observed in the groups treated with 500 and 1000 mg/kg compared with the control group (*p* < 0.05). Urea and creatinine levels were mildly but dose-dependently decreased in both the male and the female rats treated with ASME compare with their respective control groups.

#### 3.2.5. Effect of Oral Administration of *Alstonia scholaris* Extract on Serum Lipid Profile

An increment in high density lipoproteins levels (HDL) was observed in both the female (Table 7) and male rats (Table 8) treated with 500 and 1000 mg/kg b.w. of ASME compared with their respective control groups. However, the levels of total cholesterol (TC), triglycerides (TG), low density lipoproteins (LDL) and plasma glucose were not significantly different from the control group in either of the tested rat genders.

#### 3.2.6. Effect of Oral Administration of *Alstonia scholaris* Extract on Plasma Hematological Parameters

Hematological parameters of the female and male rats were examined as shown in Table 9 and Table 10, respectively. Significant dose-dependent reductions were observed in plasma hemoglobin (Hb; *p* < 0.05) levels and red blood cell count (RBC; *p* < 0.001) in the female animals treated with ASME compare with the control, with the statistical significance being limited to the groups treated with the 500 and 1000 mg/kg b.w. doses. Significant decreases in white blood cells (WBC) neutrophils (NTP) and lymphocytes counts were also observed in these two groups. Similarly, the platelets’ value was significantly lower in all of the treated groups compared to the control (0.01 < *p* < 0.05); whereas mean corpuscular hemoglobin concentration (MCHC, *p* < 0.05) was significantly lower in the groups receiving 250 and 1000 mg/kg b.w. of ASME as compared with the control group. In the male rats, Hb, platelets and WBC were significantly reduced in the rats treated with 1000 mg/kg compared with the control (*p* < 0.05). Other hematological parameters measured did not show statistically significant differences compared with the control groups.

#### 3.2.7. Histopathological Examination

**Figure 3:**
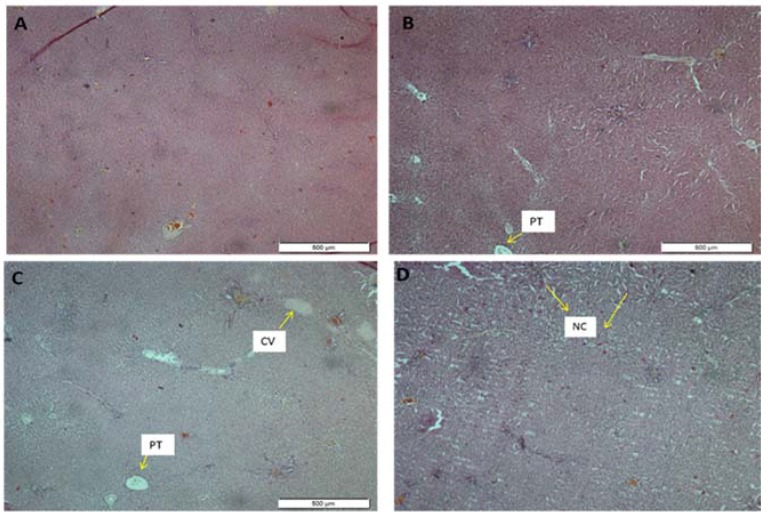
Liver sections stained with hematoxylin and eosin (H & E-stained under 40× magnification power) showing the effect of *Alstonia scholaris* methanolic extract (ASME) in a 28-day sub-acute toxicity study in female rats: (**A**) Control group; (**B**) 250 mg/kg; (**C**) 500 mg/kg and (**D**) 1000 mg/kg. Indicators: Portal Triad (PT); Central Vein (CV); Centrilobular Necrosis (NC).

**Figure 4:**
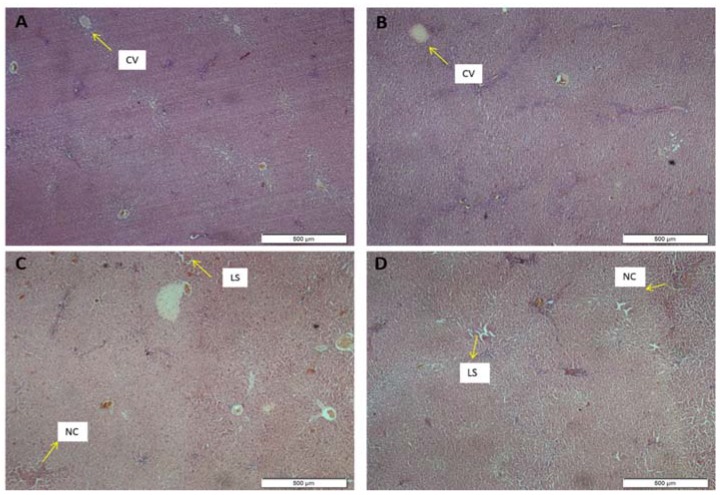
Liver sections stained with hematoxylin and eosin (H & E-stained under 40× magnification power) showing the effect of *Alstonia scholaris* methanolic extract (ASME) in a 28-day sub-acute toxicity study in male rats: (**A**) Control group; (**B**) 250 mg/kg (**C**) 500 mg/kg and (**D**) 1000 mg/kg. Indicators: Portal Triad (PT); Central Vein (CV); Centrilobular Necrosis (NC).

Histopathological examinations were performed on the liver to assess whether or not organs or tissues had been damaged. The liver appeared normal with preserved hepatic architecture. However, in both the female (Figure 3) and male (Figure 4) treated rats, slight degeneration (lesions) and centrilobular necrosis were occasionally observed in the liver, which was most expressed in the highest-dose group. Histological evaluation also showed minor change in the color of the hepatic lobules in the treated rats as compared with the control.

## 4. Discussion

Considering the numerous therapeutic potentials of *Alstonia scholaris* as an alternative medicine effective for a wide range of diseases and infections, as reported in a number of scientific papers [21], it is only pertinent that a safety profile of the plant be established as a guide for the management of its applications and usage in herbal preparations. This should serve to prevent exposing human subjects to potential toxicity-related health risks while using *A. scholaris*. Toxicity studies in appropriate animal models are commonly used to assess potential health risks in humans. Such toxicity studies assess the hazard and determine the risk level by addressing the probability of exposure to that particular hazard at certain doses or concentrations [22].

In the present study, single-dose oral administration of ASME in female rats at 2000 mg/kg b.w. had no effects on mortality, examined clinical signs, body weight or overall observation. Therefore, no acute toxicity was found in rats treated with ASME and the approximate lethal dose was determined to be higher than 2000 mg/kg. Yet, the lack of toxicity-indicative manifestations upon acute oral administration of ASME can be attributed to sub-sufficient absorption of the extract in the gastrointestinal tract, or a high first-pass metabolism rate in the liver, by which toxic components would have been converted to their harmless derivatives. Nonetheless, the knowledge gained from our acute toxicity study may serve for choosing more appropriately the test doses of *A. scholaris* extracts for later chronic or sub-chronic toxicity studies to report results of greater clinical relevance—as was the case in the present investigation.

The sub-acute toxicity study, which involved rats given ASME orally at doses of 250, 500 and 1000 mg/kg b.w., demonstrated significant changes in animal behavior, as well as significant reductions in body weight in both male and female rats at high doses (500 and 1000 mg/kg b.w.). Similarly, it was also observed that, with the exception of the lungs, no significant differences were found in the organ weights of the treated rats in comparison with the control groups. It goes without saying that a decrease in body weight may be an indicator of adverse effects [23,24]. The liver and the kidneys are target organs for toxic chemicals due to their essential functions in bodily detoxification and excretion processes. Thus, they are considered highly useful in toxicity studies because of their sensitivity to harmful compounds and their potential to predict toxicity. Toxicity-related changes in the weights of these vital organs are often accompanied by corresponding histopathological findings. Changes in the weight of the lungs have less toxicity implications due to the lungs’ limited role in the removal of harmful substances from the body [25,26]. Therefore, it could safely be claimed that the liver and the kidneys could serve as the primary target organs in investigations related to the sub-acute oral toxicity of a herbal extract.

The histological features of the liver in this study were displayed in Figure 3 and Figure 4 for the female and the male rats, respectively. The morphology of the hepatic cells in both the male and the female control groups was normal. However, in the extract-treated group, severe morphological alterations in the structure were observed, which was most expressed in the highest-dose group. Furthermore, histological evaluation showed occasional centrilobular necrosis [27]. It was also observed that the central vein in the groups treated with ASME had been enlarged, an effect which could lead to congestive hepatopathy [28].

Analysis of blood parameters in animal toxicity studies is important to report alterations in those parameters and evaluate the relative risk to the hematopoietic system when extrapolating those findings to humans [29,30]. Determining certain blood biochemical parameters and investigating major toxic effects on specific tissues, specifically the kidneys and the liver, may provide useful information regarding the mechanisms of toxicity of an otherwise safe and therapeutic agent [31]. Significant increases in the levels of some biochemical parameters, particularly ALP and AST, were observed in both the male and the female rats treated with ASME, as compared with the respective controls. ALT levels were mildly reduced in the female treated rats, but they were significantly altered in the male ones which received the doses of 250 and 1000 mg/kg b.w. Due to its distinctive abundance in the cytoplasm of liver cells, ALT has been commonly used as a marker to quantify suspected liver cell damage [32,33]. AST is more ubiquitous in nature. Besides making up 80% and 20% of the total intracellular enzymes in hepatic mitochondria and cytoplasm, respectively, it is found in the heart, skeletal muscle, kidneys, brain, pancreas and blood cells [34,35]. To state another observation, mild and statistically-insignificant increases in serum albumin, total protein, globulin and bilirubin were observed in the male, rather than the female, animals of the highest-dose group. These findings could signal mild degeneration and the presence of lesions, which was confirmed by histopathological examination of the livers of the animals in the highest-dose group. These results suggest that ASME may have altered few hepatic functions and indicate that the rats’ livers in the highest-dose group may have been injured upon sub-acute administration.

Abnormally high levels of serum creatinine, uric acid and urea are biomarkers of possible malfunction of the kidneys [36]. In this study, both urea and creatinine levels were marginally altered in both the male and the female treated rats compared with their respective controls. Nevertheless, the values were within the normal ranges of these parameters, which ruled out the possibility of precipitated abnormalities. Thus, these findings suggest that ASME does not affect the normal kidney function.

In regards to the observed hematological values, most of the values shown in the treated groups were normal in comparison with the control group. Yet, some values were significantly different from those of the control group, such as those pertaining to hemoglobin, RBC, MCV and MCHC. Reductions in these indices indicated that the extract interfered with the normal production of Hb and its concentrations within RBCs. Thus, it should be concluded that ASME may possess the potential to induce anemia [37]. Moreover, the observed reductions in WBC, including lymphocytes, monocytes, and eosinophils, and platelets counts may suggest a decline in the function of the immune system. Therefore, these results suggest that the extract has a tendency to cause anemia and immunological defects in rats, rendering the animals more vulnerable to infections. It was also evident that the hematological values were more altered in the female, rather than the male, treated rats.

The lipid profiles of the treated rats demonstrated significantly increased HDL levels, which corresponded to mild decreases in LDL levels. HDL is known to be the good cholesterol in the body as it facilitates the prevention of cardiovascular risk factors. Therefore, the observation herein further confirmed the reported antidiabetic and antihyperlipidemic activities of *Alstonia scholaris* [6], even though TC, TG and glucose levels were not significantly changed in the animals treated with ASME in this study when compared to the control group.

The death of two female rats indicate gender sensitivity of the toxic effect. However, this contradicts the previous findings by Baliga *et al.* [38] where the male rats are reported to be more susceptible to the toxic effect of the extract. Another difference is the variation in the lethal (LD_50_) dose; while the previous study [38], a sub-chronic toxicity study, showed that an oral dose of 240 mg/kg of ethanol extract of *Alstonia scholaris* bark collected in the monsoon season in India is highly toxic, in this current study, 250 mg/kg of the methanol extract was found to be non-toxic for 28-day oral administration. Ecological and environmental factors such as seasonal variation in rainfall and extraction process used on the plant sample are known to affect the phytoconstituents in any given species as much as the biological factors [39]. Thus, it is plausible that the disparity is the toxic effect may be due to differences in geographical location, collection time of the plants samples and the extraction procedure. Evidently, *Alstonia scholaris* bark extract collected during the summer period showcased a less toxic effect.

A limiting factor in this study was the lack of certainty as to whether the plant sample used could serve as a prototype of *Alstonia scholaris* species. Plant identification was based on morphological features; taxonomical data and other regional pharmacopeia information were used for validation at USM Herbarium Unit. Secondly, chemical characterization of the plant material was not conducted. Nonetheless, the toxic of *Alstonia scholaris* has previously been attributed to echitamine [38] which has been reported to exert a cytotoxic effect on cell lines [40]. Hence, although this work may serve as a template for future animal studies, and provide guidelines pertaining to the provincial use of *Alstonia scholaris* by Asian patients, considering the lack of any chemical characterization, the present results might not be comparable to any future studies utilizing this same plant species.

## 5. Conclusions

This study validated the toxic effects of *Alstonia scholaris* bark extract at the doses of 500 and 1000 mg/kg with prolonged use. The toxic effects comprised changes in the hematological compositions with end-organ damage to the liver, leading to alterations in the normal physiological functions and weakening of the immune system of the animals. Both the male and female groups treated with 250 mg/kg did not display signs of toxicity. The death of the two female rats in the high dose group may indicate that the female rats are more sensitive to ASME toxic effects than the male rats. Therefore, caution and safety measures should be taken before oral ingestion of *Alstonia scholaris* for therapeutic purposes or for other uses; and prolonged use should be discouraged and lower doses encouraged.

## Figures and Tables

**Table 1:** Organ weights (g) of the female rats in the sub-acute toxicity study of the methanolic extract of *Alstonia scholaris*. Results were expressed as the mean ± S.E.M. of 5 rats.

Female	Control (mg/kg)	*Alstonia scholaris* Extract Dose (mg/kg)		
		250	500	1000
Lungs	1.39 ± 0.11	1.32 ± 0.07	1.22 ± 0.127 *	1.27 ± 0.08 *
Heart	0.58 ± 0.03	0.53 ± 0.02	0.53 ± 0.02	0.50 ± 0.04
Liver	5.31 ± 0.24	4.94 ± 0.342	5.04 ± 0.15	5.5 ± 0.29
Pancreas	0.54 ± 0.05	0.37 ± 0.016	0.41 ± 0.046	0.52 ± 0.13
Spleen	0.52 ± 0.02	0.44 ± 0.04	0.44 ± 0.03	0.48 ± 0.03
Adrenals	0.02 ± 0.002	0.02 ± 0.002	0.02 ± 0.002	0.02 ± 0.003
Kidneys	0.57 ± 0.04	0.53 ± 0.04	0.50 ± 0.02	0.55 ± 0.03
Ovaries	0.04 ± 0.01	0.03 ± 0.002	0.03 ± 0.005	0.03 ± 0.003

Significantly different from the control, * *p* < 0.05.

**Table 2:** Organ weights (g) of the male rats in the sub-acute toxicity study of the methanolic extract of *Alstonia scholaris* bark. Results were expressed as the mean ± S.E.M. of 5 rats.

Male	Control (mg/kg)	*Alstonia scholaris* Extract Dose (mg/kg)		
		250	500	1000
Lungs	1.86 ± 0.34	1.66 ± 0.10	1.34 ± 0.21 *	1.45 ± 0.12 *
Heart	0.89 ± 0.18	0.82 ± 0.038	0.89 ± 1.001	0.8 ± 0.013
Liver	7.79 ± 1.57	6.98 ± 0.23	5.16 ± 1.17	6.9 ± 0.24
Pancreas	0.57 ± 0.12	0.48 ± 0.041	0.56 ± 0.03	0.61 ± 0.07
Spleen	0.61 ± 0.13	0.57 ± 0.06	0.55 ± 0.05	0.56 ± 0.05
Adrenals	0.02 ± 0.004	0.02 ± 0.003	0.02 ± 0.001	0.02 ± 0.001
Kidneys	0.96 ± 0.20	0.89 ± 0.02	0.82 ± 0.04	0.85 ± 0.03
Testis	1.48 ± 0.30	1.43 ± 0.04	1.31 ± 0.05	1.4 ± 0.04

Significantly different from the control, * *p* < 0.05.

**Table 3:** Plasma electrolytes values in the female rats in the sub-acute toxicity study of the methanolic extract of *Alstonia scholaris* bark. Results were expressed as the mean ± S.E.M. of 5 rats.

Female	Control (mg/kg)	*Alstonia scholaris* Extract Dose (mg/kg)		
		250	500	1000
Sodium (mmol/L)	141.73 ± 1.40	140.82 ± 1.21	136.57 ± 0.82	136.67 ± 1.23
Potassium (mmol/L)	5.20 ± 0.34	5.14 ± 0.17	5.51 ± 0.22	5.73 ± 0.23
Chloride (mmol/L)	108.65 ± 0.64	102 ± 0.38	103 ± 0.58	102.2 ± 0.46
Urea (mmol/L)	6.64 ± 0.31	6.61 ± 0.36	5.41 ± 0.24	5.53 ± 0.52
Creatinine (umol/L)	29.21 ± 1.36	26.23 ± 1.17	22.56 ± 1.09	23.11 ± 1.22
Uric acid (mmol/L)	0.07 ± 0.01	0.08 ± 0.02	0.07 ± 0.002	0.09 ± 0.03
Calcium (mmol/L)	2.48 ± 0.04	2.45 ± 0.05	2.46 ± 0.04	2.44 ± 0.03
Phosphate (mmol/L)	3.02 ± 0.12	2.94 ± 0.49	2.69 ± 0.23	2.58 ± 0.12

**Table 4:** Serum electrolytes values of the male rats in the sub-acute toxicity study of the methanolic extract of the bark of *Alstonia scholaris.* Results were expressed as the mean ± S.E.M. of 5 rats.

Male	Control (mg/kg)	*Alstonia scholaris* Extract Dose (mg/kg)		
		250	500	1000
Sodium (mmol/L)	138.67 ± 1.67	140.67 ± 0.67	141.67 ± 0.88	140.67 ± 2.03
Potassium (mmol/L)	5.50 ± 0.25	5.37 ± 0.41	4.90 ± 0.12	4.93 ± 0.28
Chloride (mmol/L)	98.67 ± 0.88	101 ± 0.58	101 ± 0.58	100.3 ± 0.88
Urea (mmol/L)	6.27 ± 0.23	7.13 ± 0.59	5.73 ± 0.18	5.50 ± 0.35
Creatinine (umol/L)	28.67 ± 1.67	27.00 ± 1.53	23.67 ± 1.20	22.00 ± 1.15
uric acid (mmol/L)	0.08 ± 0.00	0.09 ± 0.01	0.10 ± 0.003	0.09 ± 0.01
Calcium (mmol/L)	2.32 ± 0.23	2.61 ± 0.42	2.39 ± 0.27	2.43 ± 0.08
Phosphate (mmol/L)	2.92 ± 0.05	2.69 ± 0.36	2.63 ± 0.04	2.58 ± 0.04

**Table 5:** Biochemical parameters in the serum of female rats orally treated with the methanolic extract of the bark of *Alstonia scholaris*. Results were expressed as the mean ± S.E.M. of 5 rats.

Female	Control (mg/kg)	*Alstonia scholaris* Extract Dose (mg/kg)		
		250	500	1000
TP (g/L)	71.0 ± 1.53	75.67 ± 1.45	72.67 ± 0.33	71.67 ± 1.33
Albumin (g/L)	30.0 ± 1.00	30.67 ± 0.33	30.33 ± 0.67	30.00 ± 1.00
Globulin (g/L)	41.0 ± 1.53	45.00 ± 1.73	42.33 ± 0.88	41.67 ± 1.20
ALP (IU/L)	203.3 ± 12.8	284.7 ± 34.7 **	231.7 ± 19.9 *	277.67 ± 15.7 **
Bilirubin (mol/L)	2.0 ± 0.67	2.00 ± 0.67	2.33 ± 0.33	2.67 ± 0.33 *
AST (IU/L)	197.7 ± 3.93	206.67 ± 25.50	235.3 ± 22.6 **	218.67 ± 25.6 *
ALT (IU/L)	49.31 ± 3.46	39.74 ± 2.52	42.36 ± 2.04	41.34 ± 2.47

Significantly different from the control, * *p* < 0.05.; ** *p* < 0.01.

**Table 6:** Biochemical parameters in the serum of male rats orally treated with the methanolic extract of the bark of *Alstonia scholaris*. Results were expressed as the mean ± S.E.M., *n* = 5.

Male	Control (mg/kg)	*Alstonia scholaris* Extract Dose (mg/kg)		
		250	500	1000
TP (g/L)	67.21 ± 3.34	65.34 ± 4.5	70.32 ± 2.3	69.73 ± 1.52
Albumin (g/L)	28.40 ± 2.21	29.42 ± 0.46	32.37 ± 0.22	32.15 ± 1.40
Globulin (g/L)	38.24 ± 1.29	41.00 ± 3.03	39.62 ± 0.51	42.63 ± 2.50
ALP (IU/L)	113.3 ± 4.5	114.7 ± 14.7	121.7 ± 11.3 *	117.67 ± 6.9 *
Bilirubin (mol/L)	2.13 ± 0.59	2.70 ± 0.73	2.39 ± 0.47	2.84 ± 0.36
AST (IU/L)	221.32 ± 12.4	246.5 ± 21.3	257.3 ± 21.4 **	248.67 ± 16.6 *
ALT (IU/L)	49.0 ± 3.61	59.33 ± 1.86 *	54.67 ± 2.19 *	57.33 ± 3.76 *

Significantly different from the control, * *p* < 0.05.; ** *p* < 0.01.

**Table 7:** Effect of 28 days of oral administration of the methanolic extract of the bark of *Alstonia scholaris* on plasma glucose levels and lipid profiles in female rats. Results were expressed as the mean ± S.E.M. of 5 rats.

Female	Control (mg/kg)	*Alstonia scholaris* Extract Dose (mg/kg)		
		250	500	1000
TC (mmol/L)	1.77 ± 0.23	1.67 ± 0.09	1.51 ± 0.06	1.57 ± 0.12
TG (mmol/L)	0.60 ± 0.04	0.44 ± 0.02	0.59 ± 0.07	0.62 ± 0.09
HDL (mmol/L)	0.96 ± 0.11	0.93 ± 0.06	1.27 ± 0.04 *	1.18 ± 0.07 *
LDL (mmol/L)	0.53 ± 0.13	0.54 ± 0.04	0.56 ± 0.06	0.50 ± 0.04
Glucose	6.00 ± 0.87	6.07 ± 0.47	5.87 ± 0.27	5.77 ± 0.64

Significantly different from the control, * *p* < 0.05.

**Table 8:** Effect of 28 days of oral administration of the methanolic extract of the bark of *Alstonia scholaris* on serum lipid profiles and plasma glucose levels in male rats. Results were expressed as the mean ± S.E.M. of 5 rats.

Male	Control (mg/kg)	*Alstonia scholaris* Extract Dose (mg/kg)		
		250	500	1000
TC (mmol/L)	1.56 ± 0.42	1.73 ± 0.27 *	1.53 ± 0.62	1.61 ± 0.23
TG (mmol/L)	0.70 ± 0.02	0.51 ± 0.04	0.55 ± 0.03	0.61 ± 0.12
HDL (mmol/L)	0.74 ± 0.13	0.95 ± 0.07	1.02 ± 0.02 *	1.05 ± 0.18 *
LDL (mmol/L)	0.53 ± 0.16	0.54 ± 0.04	0.56 ± 0.06	0.50 ± 0.04
Glucose	5.42 ± 0.53	6.21 ± 0.36 *	5.35 ± 0.45	5.90 ± 0.38 *

Significantly different from the control, * *p* < 0.05.

**Table 9:** Serum hematological values of female rats orally treated with the methanolic extract of the bark of *Alstonia scholaris*. Results were expressed as the mean ± S.E.M., *n* = 5.

Female	Control (mg/kg)	*Alstonia scholaris* Extract Dose (mg/kg)		
		250	500	1000
Hb (g/L)	145.71 ± 1.8	147.0 ± 2.8	141.50 ± 5.4	120.40 ± 9.1 **
RBC (×10^12^/L)	788.00 ± 8.0	790.0 ± 19.8	733.83 ± 32.9 *	645.4 ± 46.7 **
PCV (L/L)	0.45 ± 0.01	0.48 ± 0.01	0.45 ± 0.02	0.39 ± 0.03
MCV (fL)	56.57 ± 0.46	61.17 ± 1.51	61.33 ± 2.40	60.00 ± 1.10
MCH (pg)	18.29 ± 0.31	18.67 ± 0.33	19.33 ± 0.42	18.80 ± 0.20
MCHC (g/L)	327.29 ± 6.94	305.17 ± 4.6 **	318.50 ± 17.9	311.0 ± 4.5 *
RDW (%)	17.27 ± 0.61	17.45 ± 0.86	16.07 ± 0.93	16.18 ± 0.60
WBC (×10^3^/mm^3^)	53.43 ± 11.81	54.0 ± 12.72	38.67 ± 6.46 *	36.80 ± 3.85 *
NEUTRO (×10^9^/L)	12.14 ± 2.59	12.0 ± 2.21	6.17 ± 0.87 *	6.40 ± 0.93 *
LYMPH (×10^9^/L)	37.00 ± 8.20	38.17 ± 10.07	41.00 ± 7.04	27.80 ± 3.22
MONOS (×10^9^/L)	1.71 ± 0.61	1.83 ± 0.40	1.83 ± 0.17	1.80 ± 0.58
EOSINO (×10^9^/L)	1.67 ± 0.42	4.00 ± 1.55	1.00 ± 0.01	1.25 ± 0.22
PLT (×10^9^/L)	923 ± 50	672 ± 118 **	760 ± 106 *	687 ± 104 **

Significantly different from the control, * *p* < 0.05.; ** *p* < 0.01.

**Table 10:** Serum hematological values of male rats orally treated with the methanolic extract of the bark of *Alstonia scholaris*. Results were expressed as the mean ± S.E.M., *n* = 5.

Male	Control (mg/kg)	*Alstonia scholaris* Extract Dose (mg/kg)		
		250	500	1000
Hb (g/L)	153.3 ± 7.3	151.67 ± 1.86	149.67 ± 1.45	143 ± 3.06 *
RBC (×10^12^/L)	8.54 ± 0.05	8.96 ± 0.04	8.78 ± 0.16	8.3 ± 0.43
PCV (L/L)	0.45 ± 0.02	0.45 ± 0.01	0.44 ± 0.01	0.42 ± 0.01
MCV (fL)	52 ± 2.52	50 ± 0.58	50 ± 1.53	51 ± 3.21
MCH (pg)	18 ± 1.00	17 ± 0.01	17.33 ± 0.67	17.67 ± 0.88
MCHC (g/L)	345.7 ± 3.4	338 ± 4.5	340.7 ± 2.3	340.3 ± 5.6
RDW (%)	17.77 ± 0.7	19.47 ± 0.37	21.13±2.04	21.07 ± 4.09
WBC (×10^3^/mm^3^)	10.4 ± 1.12	9.37 ± 2.42	9.7 ± 1.76	8.87 ± 2.3 *
NTP (×10^9^/L)	2.2 ± 0.65	2.00 ± 0.53	1.83 ± 0.58	2.53 ± 0.97
LYMPH (×10^9^/L)	7.4 ± 0.45	6.97 ± 1.7	7.43 ± 1.09	6.07 ± 1.98
MONOS (×10^9^/L)	0.67 ± 0.13	0.3 ± 0.15	0.33 ± 0.12	0.17 ± 0.07
EOSINO (×10^9^/L)	0.1 ± 0.01	0.07 ± 0.03	0.1 ± 0.01	0.07 ± 0.03
PLT (×10^9^/L)	928 ± 55	989 ± 67	1121 ± 19 *	1230 ± 41 *

Significantly different from the control, * *p* < 0.05.
